# Insights from a single centre implementation of a digitally-enabled atrial fibrillation virtual ward

**DOI:** 10.1371/journal.pdig.0000475

**Published:** 2024-03-20

**Authors:** Keenan Saleh, Jasjit Syan, Pavidra Sivanandarajah, Michael Wright, Sarah Pearse, Jodian Barrett, James Bird, Grant McQueen, Sadia Khan

**Affiliations:** 1 National Heart and Lung Institute, Imperial College London, London, England; 2 Chelsea & Westminster NHS Foundation Trust, London, England; 3 Northwest London Virtual Hospital, Imperial College Healthcare NHS Trust, London, England; Washington University in Saint Louis, UNITED STATES

## Abstract

Atrial fibrillation (AF) is the most prevalent cardiac arrhythmia and poses a significant public health burden. Virtual wards are a novel approach utilising digital solutions to provide hospital-level care remotely; their rollout has become a key priority for the UK National Health Service to expand acute care capacity. We devised and implemented a digitally-enabled AF virtual ward to monitor patients being established onto medical therapy following an AF diagnosis or an AF-related hospitalisation. Patients were onboarded either as outpatients to avoid admission or on discharge after an acute AF hospitalisation. Remote monitoring was undertaken using a clinically validated photoplethysmography-based smartphone app. Over a 1–2 week period, patients performed twice daily measurements of heart rate and rhythm and provided corresponding symptoms. A traffic light system guided frequency of telephone assessments by specialist practitioners. Red flag symptoms or abnormal heart rate parameters prompted an urgent care escalation. We report our experience of the first 73 patients onboarded to the AF virtual ward from October 2022 to June 2023 (mean age 65 years, median 68 years, IQR range 27–101 years; 33 females). Thirty-nine (53%) patients had red flag features requiring care escalation, of whom 9 (23%) were advised to attend ED (emergency department) for urgent assessment, 10 (26%) attended for expedited review and 14 (36%) required medication changes. By 3 months post-monitoring, only 3 patients (4%) had re-attended ED with an arrhythmia-related presentation. Virtual ward patients had an average 3-day shorter inpatient stay (mean duration 4 days) compared with AF patients hospitalised prior to virtual ward implementation (mean duration 7 days). Overall, 22 arrhythmia-related readmissions were prevented via the virtual ward model. In this study, we present a novel implementation of a digitally-enabled virtual ward for the acute management of patients with newly diagnosed or poorly controlled AF. Our pilot data indicate that this model is feasible and is potentially cost-effective. Further longitudinal study is needed to definitively evaluate long-term clinical utility and safety.

## Author summary

Atrial fibrillation (AF) is a heart rhythm abnormality associated with labile heart rates. If AF patients’ heart rates remain rapid and uncontrolled, their health may deteriorate requiring acute hospitalisation. AF patients are often admitted for monitoring while their heart rate remains elevated until control is achieved with medications. Virtual wards can provide these monitoring capabilities remotely alongside regular clinical review, which can enable these patients to be cared for from their own homes. This approach seeks to improve the efficiency of care delivery and reduce healthcare costs while maintaining patient safety. We created an AF virtual ward pathway across our hospital trust, utilising patients’ own smartphones, a patient-facing digital health record platform and a centralised remote monitoring hub. We share our experience of the implementation of this pathway and highlight the benefits in terms of earlier recognition of patient deterioration, reduction in the number of patients being rehospitalised due to their AF and potential cost savings due to reduced length of hospital inpatient stay and rates of readmission.

## Introduction

Atrial fibrillation (AF) is the most common cardiac arrhythmia in the UK. Public Health England estimates that ~1,300,000 people are living with AF in the UK [1] and projections indicate that this could reach as high as 1,850,000 by 2060 [2]. Treatment is targeted towards the prevention of AF-related complications, such as heart failure and stroke, and the improvement of symptoms and quality of life. Timely evaluation is key to ensure that oral anticoagulation is promptly commenced in high-risk patients, as well as to initiate and assess response to pharmacological therapies.

In response to the COVID-19 pandemic and the resultant care backlogs, there has been increasing recognition of the transformative potential of digital technologies in healthcare delivery [3]. Remote monitoring can now be readily achieved for a variety of healthcare conditions using real-time data from smartphones, smartwatches, and other wearables. Integrated with novel digital platforms, these remote monitoring technologies have enabled the creation of virtual ward environments; this has allowed patients with chronic conditions to be managed safely from home with enhanced support from their specialist teams and has promoted more accessible, efficient, and personalised care [4]. Models of care utilising remote monitoring also have the potential to streamline care delivery, generate cost savings, mitigate health inequalities and provide enhanced vigilance against deterioration in patients with dynamic health conditions. Pertaining to cardiac arrhythmias, a consensus statement by the Heart Rhythm Society and European Heart Rhythm Association has highlighted the growing role of remote monitoring to enable earlier detection of important parameter changes which may portend health deterioration [5].

To further improve acute care delivery and capacity, the National Health Service (NHS) in England is actively encouraging the expansion and rollout of virtual wards which can enable early supported discharge and provide alternatives to admission [6]. This national focus has arisen in response to escalating inpatient bed pressures and service constraints within the NHS and the wider healthcare system. Virtual ward systems are associated with improved care outcomes and lower healthcare costs [7]. Additional benefits include reductions in patient transport and in the carbon footprint associated with acute hospitalisations and vehicular travel [8].

Locally, we have actively developed digital tools to improve cardiovascular care. We have utilised a clinically validated smartphone app (FibriCheck), incorporating photoplethysmography recordings and proprietary algorithms to screen for AF in patients following cryptogenic stroke. We sought to evaluate additional uses of this technology as part of an AF virtual ward workflow, which could be piloted alongside pre-existing virtual ward pathways for COVID-19 infection, heart failure, frailty and COPD.

We postulated that leveraging remote monitoring technology and digital platforms would enhance the care of patients with newly diagnosed or poorly controlled AF in the short term while rate control is being established, particularly while patients are awaiting outpatient review by a cardiology specialist. Therefore, we embarked on a service improvement project, aiming to develop a virtual ward model of care for AF patients to support admission avoidance following a new AF diagnosis and facilitate early supported discharge after an AF-related hospitalisation.

## Methods

### Overview

We initially undertook a retrospective analysis of AF presentations to our emergency department, from March 2022 – September 2022. Our results suggested that a significant proportion of patients could be more optimally managed in an outpatient setting with remote monitoring to reduce length of stay, improve flow through the hospital and produce potential cost savings with reduced bed occupancy. We therefore developed and implemented an AF virtual ward across 2 North West London hospital sites (West Middlesex Hospital and Chelsea & Westminster Hospital). This work was approved by NHS North West London and the trust’s governance processes for service improvement and innovation and hence formal ethical approval was not sought. Here, we prospectively evaluated clinical outcomes in patients enrolled onto the AF virtual ward from November 2022 to June 2023. Patients were onboarded via two streams: either as outpatients following a referral from the SDEC (Same Day Emergency Care unit) or cardiology clinic, or alternatively while inpatient at the point of hospital discharge. Patients provided self-measurements of their heart rate and rhythm, as well as their contemporaneous symptoms, via a smartphone app. Daily remote monitoring was undertaken by a centralised hub staffed by trained remote monitoring practitioners with cardiologist oversight. Patients were remotely monitored via the virtual ward for up to 2 weeks in line with local virtual ward policy, based on NHS England guidance [6].

### Study population

Patients were eligible for virtual ward enrolment if the following criteria were met:

1. ≥18 years old
2. New diagnosis of AF OR pre-existing AF with poor rate control (atrial flutter was also included as this is often concomitant with AF and may be managed in the same way)
3. Haemodynamically stable with no other active condition requiring inpatient management
4. Patient or carer owns a smartphone capable of downloading the remote monitoring application and able to engage in twice daily remote monitoring
5. Patient or carer able to speak / understand English

### Remote monitoring application

**Fig 1:**
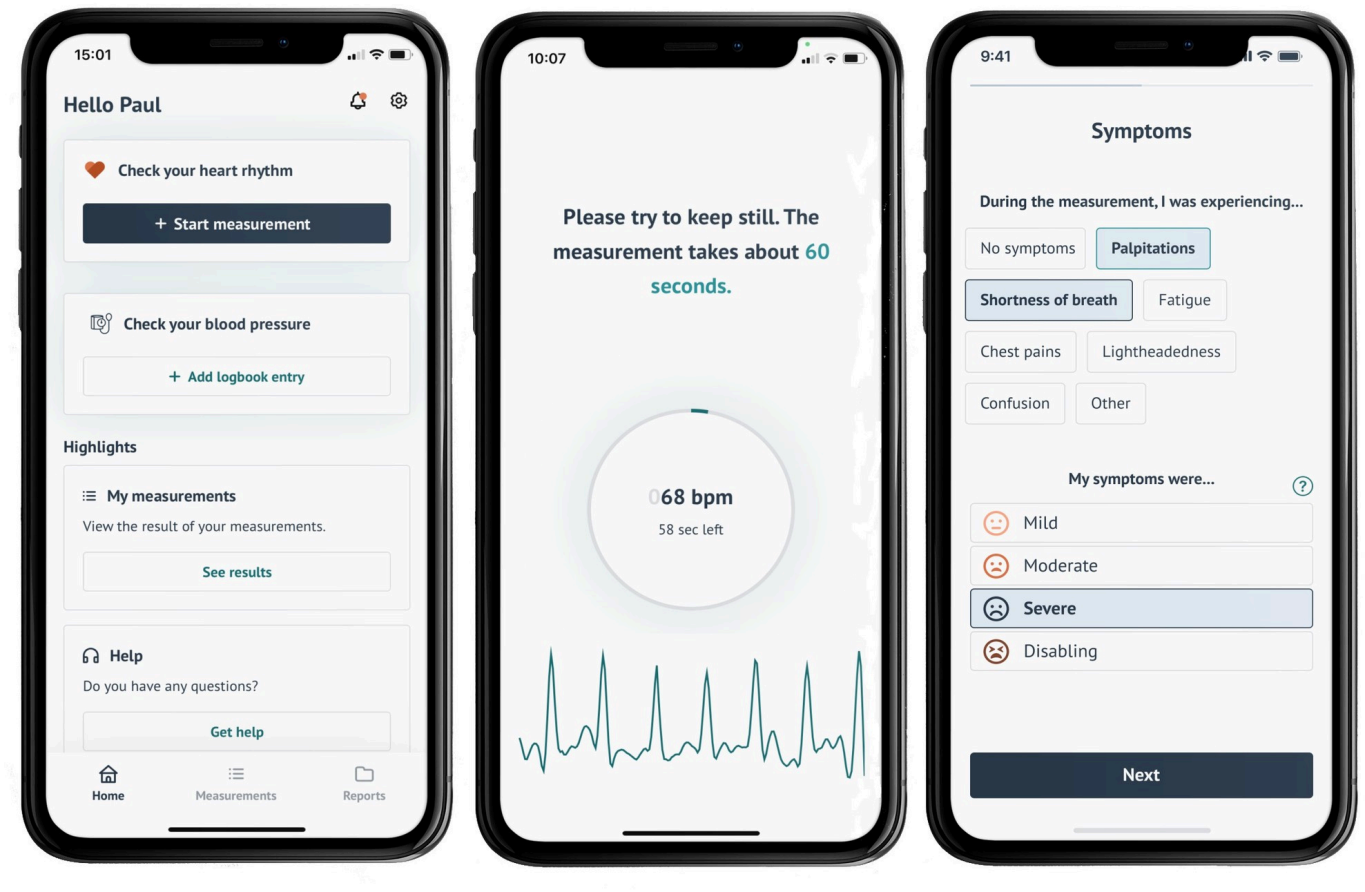
FibriCheck smartphone interface.

Patients or their carers were asked to download the FibriCheck application onto their smartphone by a member of the patient onboarding team – either a general nurse working on the virtual ward programme or a member of the remote monitoring team. FibriCheck is a smartphone-based remote monitoring application which utilises in-built photoplethysmography and camera functionalities within the smartphone to provide measurements of heart rate and rhythm (Fig 1). The FibriCheck application has attained regulatory medical device certification from the European Medicines Agency (CE-marked class IIa) and clearance from the US Food and Drug Administration. Highly accurate and clinically validated rhythm detection algorithms are applied in-app to the signal measurements, which are subsequently verified by clinical experts from the FibriCheck team; this enables the discrimination of different heart rhythms such as sinus rhythm, atrial fibrillation, or ectopic beats [9]. Patients are also able to provide their symptoms and severity score within the application. Training and assistance was provided for downloading the application and performing measurements by trained clinicians. A free activation code was provided for 7-day access to the FibriCheck platform. Patients were asked to provide measurements and symptom entries twice daily over the monitoring period, but additional ad hoc measurements could also be provided when patients experienced AF-related symptoms. Where technical issues precluded the provision of heart rate measurements via the FibriCheck app, patients were able to use alternative consumer PPG devices if available or were provided with a pulse oximeter.

### Digital health record platform

**Fig 2:**
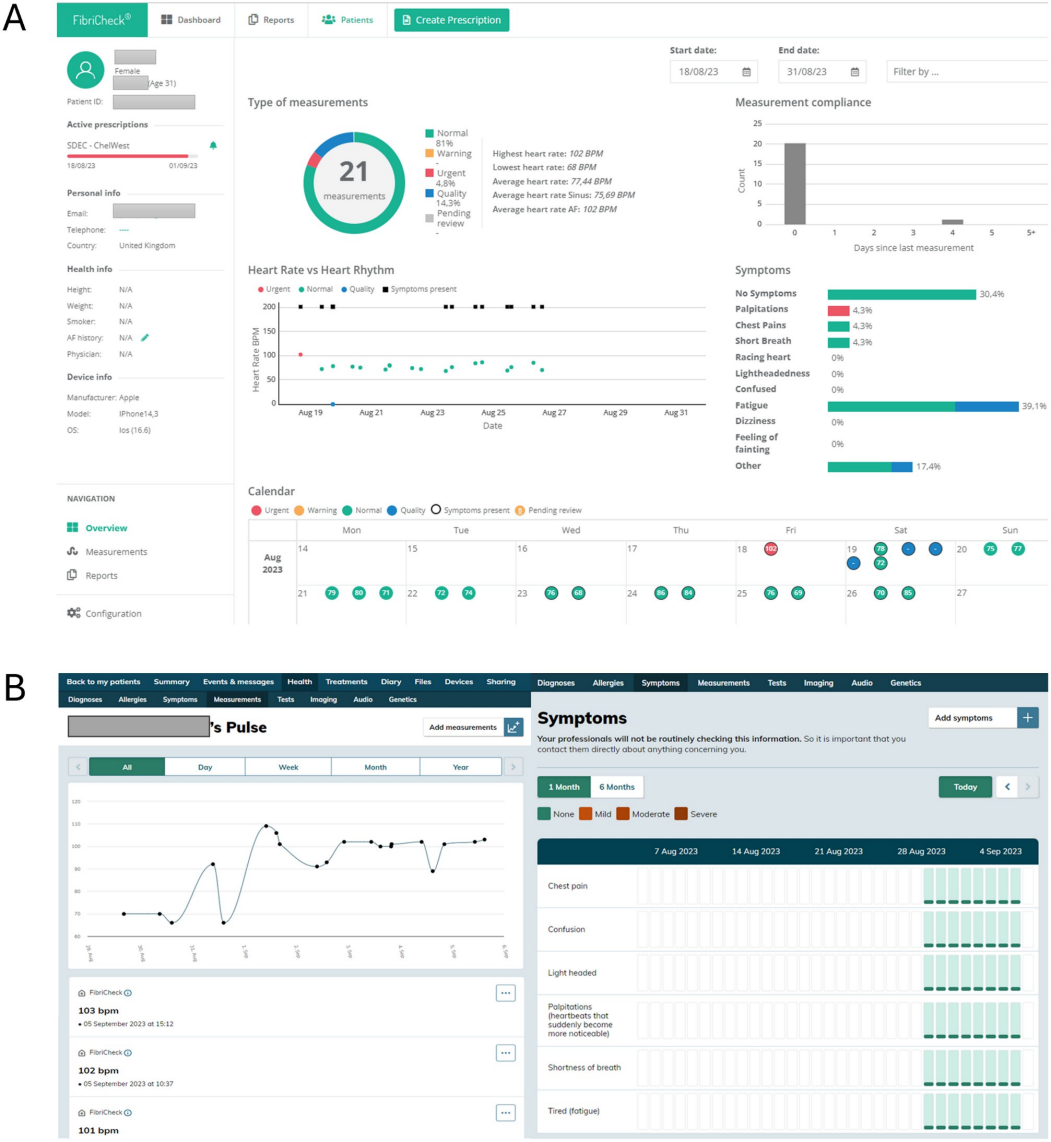
Remote monitoring infrastructure. (A) FibriCheck clinical dashboard and (B) Patients Know Best platform.

Patient measurements and symptom data from the FibriCheck app were securely uploaded from patients’ mobile phones through an integration with a secure web-based digital platform called Patients Know Best. This platform enables patients to access their personal health record and serves as an interface between patients and healthcare providers across the North West London region. An AF care plan was created for each patient (S1 Appendix) which included an overview of their measurements and symptom data, as well as advice on AF self-management, app troubleshooting, safety netting recommendations and contact information for further support. The Patients Know Best platform is compliant with UK GDPR requirements and has been certified against the Cyber Essentials standard for cyber security. The Patients Know Best platform and FibriCheck clinician-facing dashboards (Fig 2) were used as the interfaces for the remote monitoring team.

### Remote monitoring hub

**Fig 3:**
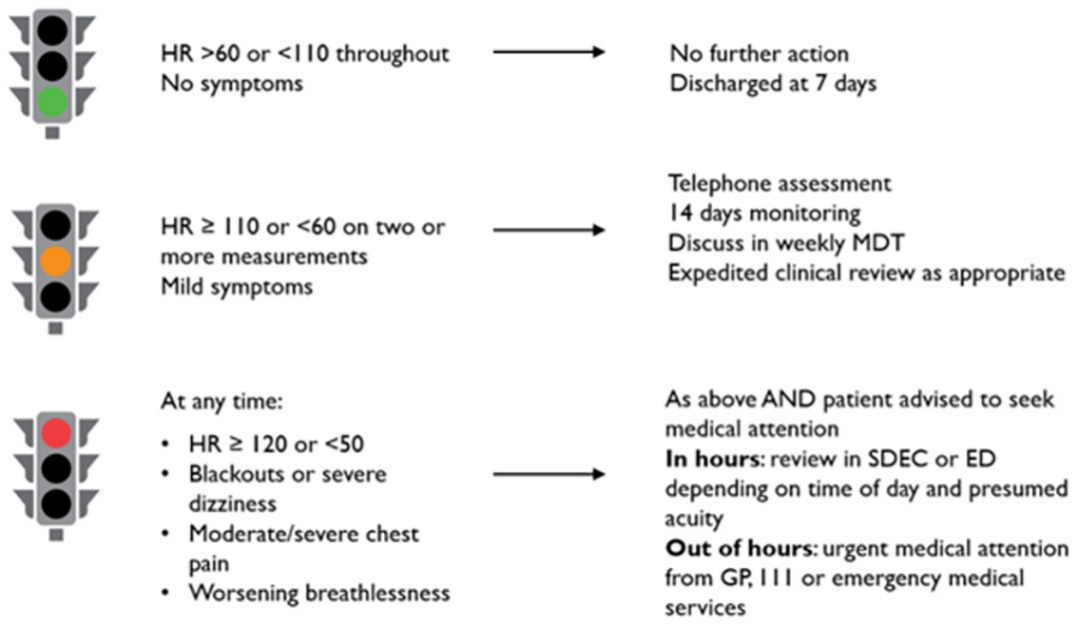
Diagram of the red-amber-green traffic light system for care escalation. SDEC–same day emergency care unit, ED–emergency department.

Remote monitoring was undertaken via a centralised hub, staffed by experienced remote monitoring practitioners from a nursing and allied health professional background. The remote monitoring hub supports several virtual ward workstreams, including atrial fibrillation, COVID-19, COPD, chest pain, heart failure and inflammatory bowel disease. The Patient Knows Best dashboard containing patient measurement and symptom data was reviewed twice daily as part of the remote monitoring process. Telephone contacts were made at the initiation of monitoring, as well as in the event of abnormal parameters, missed measurements and at the end of the monitoring interval. A red-amber-green traffic light system was used to inform telephone triage and escalation decisions, including whether patients required medication changes, expedited clinical review in SDEC or urgent clinical review in ED (Fig 3). The on-call cardiology registrar or consultant provided clinician oversight and decision-making support as required. Patients were able to contact the remote monitoring hub during working hours 8am-8pm daily to discuss their symptoms, seek medical assistance or for troubleshooting assistance. Outside of these hours, in the event of red flag symptoms or parameters, patients were recommended to seek urgent medical attention via the 111 or 999 emergency helplines.

### Patient follow up

All current virtual ward patients were discussed at a weekly multidisciplinary team (MDT) meeting, including the cardiology team and remote monitoring practitioners. This forum facilitated discussion regarding the follow up duration under the virtual ward and long-term management plans on a case-by-case basis, as well as flagging patients who may require an early specialist clinic review or referral for DC cardioversion. Where prolonged virtual ward follow up was recommended, a further 7-day activation code was provided to patients by the remote monitoring hub team.

### Integration of heart rhythm data

**Fig 4:**
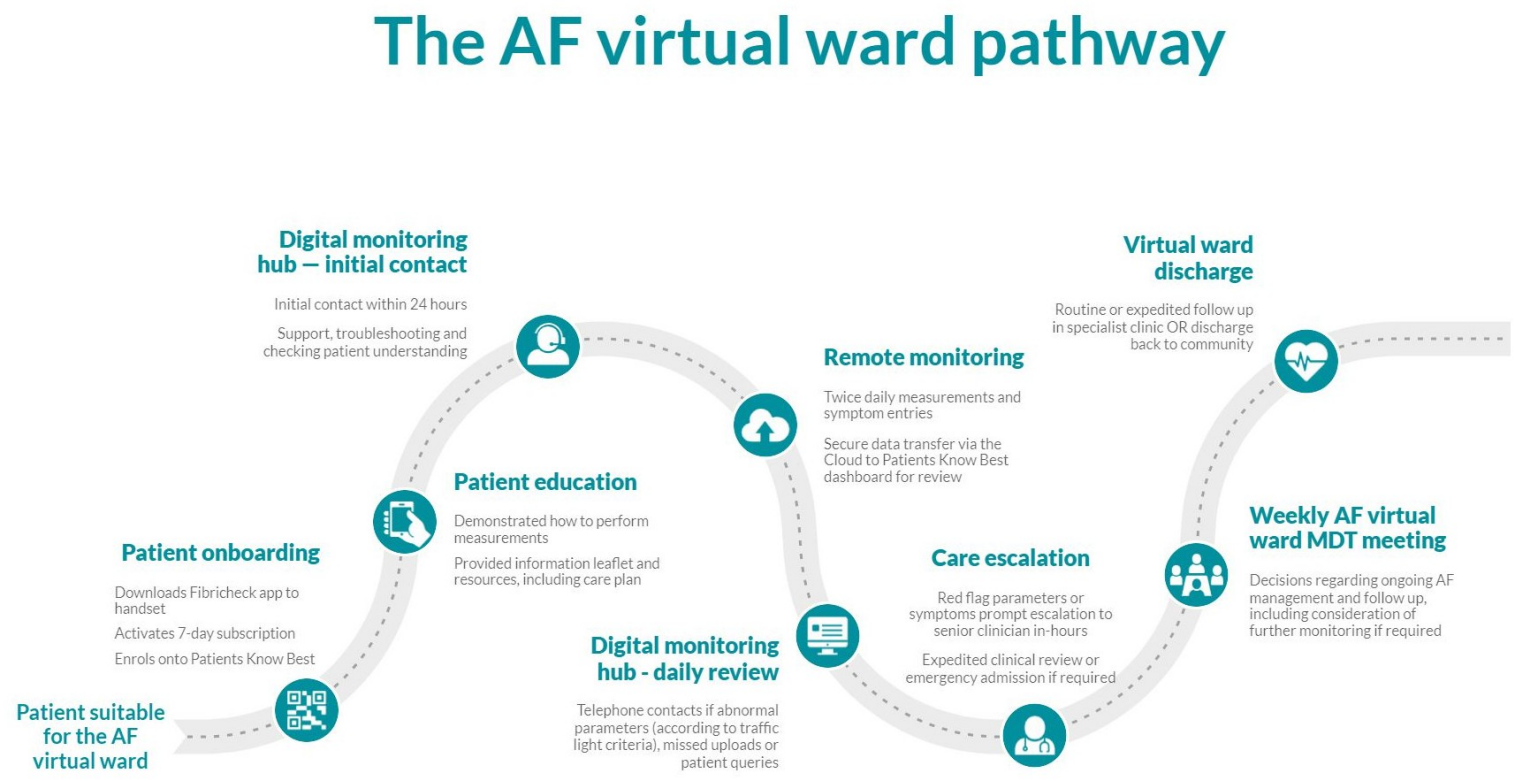
Diagram of the AF virtual ward pathway.

A 7-day patient summary report was produced via the FibriCheck app and uploaded to the patient’s electronic health record, synthesising patient measurements and symptom data over the monitoring period with algorithmically derived heart rhythm data. These FibriCheck summary reports were available for review by the cardiology team and other clinicians to provide information regarding adequacy of rate control, frequency of symptoms and rhythm-symptom correlation to guide ongoing AF management at subsequent specialist clinic visits. A summary of the pathway can be found below (Fig 4).

### Outcome variables

Anonymised data was extracted from the electronic health record as well as from the FibriCheck platform. Clinical outcomes included length of inpatient stay prior to virtual ward enrolment, ED attendances during virtual ward follow up, expedited reviews in SDEC during virtual ward follow up, changes to AF drug regimens during virtual ward follow up, and AF-related ED re-attendances or readmissions within 3 months from virtual ward discharge. Other quantitative metrics included patient compliance with measurements, measurement signal quality, and heart rate and rhythm measurements recorded during the monitoring interval.

Patient satisfaction was assessed via the Friends and Family Test, whereby patients were invited to submit feedback regarding their experience of the AF virtual ward service via an anonymous postal questionnaire. Patients were surveyed on their overall virtual ward experience when compared to inpatient treatment, how safe they felt with remote monitoring, how easily they could obtain support and advice, their perception of frequency of telephone contacts, and the ease of use of the digital monitoring platform.

### Statistical analysis

This work was undertaken as a service improvement project rather than a research study, therefore formal power calculations were not performed as part of the methodology. Descriptive statistics have been used where applicable.

### Ethics statements

This work was undertaken as a service improvement project rather than as a research study. Appropriate approval and registration was obtained under the auspices of the London North West Virtual Ward programme and via local governance processes. Clinical data was anonymised at the outset by the clinical team and no patient-identifiable information was used in the data analysis. Patient consent was not required.

## Results

### Audit of acute AF-related hospitalisations

We initially undertook a retrospective analysis of AF presentations to our emergency department over the 6-month period preceding implementation of the AF virtual ward (March 2022 – September 2022). We determined that the mean length of stay of hospitalised AF patients was 7 days. Patient turnaround was influenced by several factors, including inadequacy of rate control, patients awaiting further inpatient tests or treatments, concomitant medical conditions requiring ongoing acute care and social concerns. Furthermore, around 9% of patients hospitalised with AF as their primary diagnosis subsequently re-presented to the emergency department (ED) within 1 month of discharge with an arrhythmia-related presentation, namely AF with rapid ventricular rate, AF symptom intolerance or symptomatic bradycardia. The results are summarised in Table 1 below.

**Table 1:** Results summary of local audit of AF hospitalisations.

Patients presenting to ED with AF as primary diagnosis	n = 607
Patients admitted–no. (%)	389 (64%)
Length of inpatient stay (days)	
- Mean	7
- Median	3
- IQR	1–9
Arrhythmia-related ED reattendances within 1 month of discharge–no. (%)	57 (9%)
- AF with rapid ventricular response	45 (79%)
- AF symptom intolerance	10 (18%)
- Medication-related bradycardia	2 (3%)
Mean days to ED reattendance from discharge	10
Arrhythmia-related readmissions–no. (%)	20 (35%)
- Mean length of inpatient stay on readmission (days)	5.5

AF–atrial fibrillation; ED–emergency department.

### Implementation of the AF virtual ward

Seventy-three patients were onboarded onto the AF virtual ward from October 2022 to June 2023. The mean age of onboarded patients was 65 years old, with an interquartile range from 59 to 75 years old. The oldest onboarded patient was 101 years old. Forty patients (55%) were male. Thirty patients (41%) were newly diagnosed with atrial fibrillation on the index admission or hospital attendance before referral to the AF virtual ward. Forty-two inpatients (58%) were referred directly to the virtual ward to facilitate early supported discharge, having been admitted for a mean of 4 days; when compared with hospitalised AF patients from the 6-month period prior to virtual ward implementation, as previously referenced, this equated to a mean saving of 3 bed days per patient.

The remaining 31 patients enrolled to the AF virtual ward were referred from either the SDEC unit, cardiology outpatients or directly from ED where admission could be avoided. The mean heart rate at the time of referral to the AF virtual ward was 87bpm (beats per minute), with 21 patients (29%) being referred with a heart rate above 100bpm. Patient demographics are presented in Table 2 below.

**Table 2:** Patient demographics.

Characteristic	AF virtual ward enrolments (n = 73)	AF-related hospitalisations (n = 607)
Time period	October 22 –June 23	March 22—September 22
Female sex–no. (%)	33 (45%)	298 (49%)
Age (years)		
- Mean	65	71
- Median	68	73
- IQR	59–75	63–81
New onset AF–no. (%)	30 (41%)	324 (53%)
Ethnicity–no. (%)		
- Caucasian	55 (75%)	418 (69%)
- South Asian	10 (14%)	68 (11%)
- East Asian	2 (3%)	11 (2%)
- Afro Caribbean	2 (3%)	23 (4%)
- Other	4 (5%)	47 (8%)
- Unknown	-	40 (7%)
Referral source–no. (%)		
- Wards	42 (58%)	-
- SDEC unit or cardiology outpatient clinic	23 (32%)	-
- Emergency department	8 (11%)	-
Mean heart rate at time of referral (bpm)	87	-
Monitoring duration–no. (%)		
- 1 week	24 (33%)	-
- 2 weeks	49 (67%)	-
Rhythm during monitoring–no. (%)		
- Persistent AF	30 (41%)	-
- Paroxysmal AF	26 (36%)	-
- Sinus rhythm	17 (23%)	-

AF–atrial fibrillation; bpm–beats per minute; ED–emergency department.

Twenty-four patients (33%) were discharged after 1 week of remote monitoring, whereas 49 patients were monitored for the full 2-week period. Remote monitoring was uneventful in 34 patients (47%). In the remaining 39 patients, 32 patients recorded abnormal heart rate parameters, 15 patients recorded at least moderate-severe symptoms, and 8 patients recorded a combination of both over the course of the remote monitoring.

Among these 39 patients who triggered care escalation to the cardiology team, 13 had changes made to their AF drug regimen, 11 had an expedited or next day review in the SDEC unit or cardiology clinic and 9 patients were appropriately advised to attend ED or seek urgent medical attention. Of the 9 patients who were advised to seek urgent medical attention, 7 were admitted with AF with fast ventricular rate, 1 was admitted with decompensated heart failure and 1 was diagnosed with tachybrady syndrome and underwent permanent pacemaker implantation. Two additional patients self-presented to ED of their own volition due to concerns regarding their symptoms or heart rate parameters and were both reassured and discharged the same day without any treatment changes. There were no adverse events or deaths seen in our patient cohort.

Overall, 93% (68/73) of patients were compliant with remote monitoring. Although several patients required additional prompting to provide measurements, only 5 patients did not provide measurements consistently or refused contacts from the remote monitoring hub.

Technical issues were encountered by some of the virtual ward patients. Eight patients were unable to provide measurements via the FibriCheck app, 6 of whom were provided with a pulse oximeter instead to perform heart rate measurements. Furthermore, 1 patient used their personal Fitbit device, and 1 patient used their personal Apple watch device. Troubleshooting issues with the FibriCheck application and integration with the Patient Knows Best platform were all successfully resolved following telephone assistance from the remote monitoring hub.

Patient feedback received by the remote monitoring hub was positive. In total, 43 responses were returned with 7 pertaining to the AF virtual ward stream. All respondents indicated that they felt better looked after on the AF virtual ward then had they remained as inpatients in hospital. All respondents reported feeling safe while undergoing virtual ward monitoring and felt able to seek help or advice when required. All patients perceived that they were contacted at regular intervals, with just over a quarter of surveyed patients indicating that this was more frequently than they felt necessary. In terms of user friendliness of the remote monitoring platform, 86% of patients reported that the app was easy to use.

At discharge from the virtual ward, follow up was arranged in a cardiology or arrhythmia clinic for 65 patients (89%) and the remainder were discharged back to the community. Nine patients (12%) were directly referred for an outpatient DC cardioversion following discussion in the AF virtual ward MDT. By 3 months following discharge from the AF virtual ward, there were 3 ED re-attendances with an arrhythmia-related presentation (4%); all 3 patients presented with AF with rapid ventricular rate—1 had run out of their rate control medication, 1 had been poorly compliant with remote monitoring and 1 had had a recurrence of AF the day after an elective DC cardioversion, following which their rate control medications had been stepped down. All three patients who re-attended ED were admitted for a median duration of 3 days.

## Discussion

In this study, we share our early experiences of the implementation of an AF virtual ward to remotely monitor patients being established on treatment following an acute presentation. This intervention facilitated the early detection of health deterioration in 9 of the 73 patients, resulting in appropriate recommendations to seek urgent medical attention or call emergency care services. Over a longer 3-month time span following completion of remote monitoring, only 3 patients re-attended with an arrhythmia-related presentation, and a clear cause can be isolated in each case. Our data demonstrates the safety and feasibility of a virtual ward model for AF care, with an overall clinical benefit realised for both admission avoidance and early supported discharge of inpatients.

Based on these data it is also likely that our virtual ward model of care is cost effective. AF virtual ward patients had a 3-day shorter mean inpatient stay overall when compared to AF patients hospitalised prior to virtual ward implementation. The average daily cost per inpatient at our institution was calculated to range between £183–£330 per bed day (the lower value being a basic cost-out for provision of the bedspace and the higher value accounting for additional costs encompassing more aspects of hospital care). Maintaining AF virtual ward occupancy between 10–15 patients a month, in keeping with current occupancy levels, suggests lower and higher cost saving estimates range between £5490–£14,850 per month through savings of 30–45 hospital bed days.

Further cost savings can be attributed to prevention of readmissions. At 3 months, only 3 patients had been readmitted with an AF-related hospitalisation following remote monitoring via the AF virtual ward. If 35% of AF admissions are subsequently readmitted with an issue related to their AF (Table 1), the virtual ward saved 22 out of the 25 readmissions to hospital. Assuming a mean length of inpatient stay on readmission of 5.5 days following an AF hospitalisation (Table 1), this would equate to a projected total of 121 bed days saved and a cost saving of £22,143–£39,930 during the AF virtual ward pilot.

The care model remains economical when accounting for expenditure related to the AF virtual ward. The remote monitoring hub staffing is 2.6 full time equivalent (FTE) band 6 nurses, which encompasses all virtual ward workstreams. The AF workstream of the remote monitoring hub is managed by 1 FTE band 6 nurse, equating to total monthly gross salary expenditure between £2949.25–£3551.42 per month according to national NHS pay scales for 2023/24 [10]. Medical oversight is provided by cardiology middle grades with an on-call commitment at 0.2 FTE and cardiology consultants at 0.1 FTE, totalling approximately £2583 per month. Non-pay expenditure is very low given the fact that the approach only relies on smart phone monitoring and provision of reusable pulse oximeters, access codes and licenses for the remote monitoring application total no more than £400 per month. Additional overhead costs are institution-dependent but are minimal overall due to the virtual ward care model. Thus, when accounting for healthcare utilisation costs, we estimate savings achieved via the virtual AF pathway to be at least £20,000 per month.

Most recently, UK colleagues in Leicester have developed a similar virtual ward model for AF using a digital remote monitoring platform [11]. These patients received more intensive monitoring, and were provided with single lead ECG recorders, pulse oximeters and Bluetooth-integrated blood pressure machines. In this study, we demonstrate that a virtual ward can be successfully and readily employed at relatively low cost, without the purchase of additional expensive devices, suggesting wide applicability of AF virtual wards outside of specialist cardiac centres. Additionally, by establishing a regional remote monitoring hub we have been able to support several concomitant virtual ward pathways in tandem to improve resource efficiency without impacting care quality. In contrast to the Leicester AF virtual ward, patients onboarded to our AF virtual ward all underwent a rate control treatment strategy. This reflects contemporary practice at non-specialist cardiology centres [12], where AF management is led by general cardiologists prior to onward referral to a specialist electrophysiology centre. Thus, our virtual ward model is more generalisable to most cardiology centres without an electrophysiology service.

We elected to use a smartphone-based remote monitoring application which captured PPG signals from the handset to generate heart rate and rhythm measurements. While the European Society of Cardiology guidelines do not currently endorse the use of PPG-based technologies for AF diagnosis, particularly in view of the high false positive rate [13,14], growing evidence has indicated a role for PPG monitoring in different modalities to detect AF in patients with a known AF diagnosis [15,16]. The main reasons we utilised a smartphone-based remote monitoring approach are the relative ubiquity and broad accessibility of smartphones, the low cost, convenience and the potential scalability of our solution. The FibriCheck smartphone application has itself been evaluated as part of a multi-centre international trial for the remote management of AF alongside teleconsultations during the COVID-19 pandemic, with 94% of patients reporting that the app was easy to use in participant surveys [17].

Technical challenges with producing adequate PPG signals from patient smartphones formed one of the main barriers to implementation of the virtual ward. PPG signals have inherent limitations given their susceptibility to movement artefact and noise, as well as requiring good skin contact to produce a consistent trace [18]. This was further compounded by the fact that the finger positioning to derive an adequate PPG trace varied between handsets, with some more difficult to achieve than others and a clear learning curve required to produce an adequate trace.

To address these issues, we utilised the expertise of digital technology specialist nurses trained to onboard patients (and/or their carer) and familiarise them with the digital elements of the virtual ward and demonstrate how to perform measurements on their personal handset. For patients who did not have the manual dexterity to produce these measurements or did not have adequate support to perform these measurements in their home environment, a pulse oximeter was offered instead and patients were contacted to take a reading and provide symptom updates. The use of a pulse oximeter also enabled those patients without a compatible smartphone to be onboarded onto the virtual ward, promoting digital inclusivity. We recognise that pulse oximeter measurements of heart rate may not be validated for atrial fibrillation and this will continue to be an area for further work as these models develop.

Most patients were motivated to engage in remote monitoring, with 93% of patients performing regular measurements, although prompting was sometimes required through telephone contacts by the remote monitoring team. A consistent theme from received feedback was that the provision of remote monitoring enabled patients to feel safer in their own homes, better supported and more empowered to manage their health. We did encounter a small number of patients with an aversion or anxiety towards digital technologies, which is in keeping with feedback from similar AF virtual ward models [11,19].

We anticipated varying levels of digital literacy in our patient cohort but were still able to successfully onboard patients from an older and generally less digitally familiar demographic, with the oldest patient being 101 years old. This was achieved through the heavy focus on patient education to guide the use of the digital health record platform and smartphone remote monitoring app, by the onboarding team as well as the remote monitoring hub practitioners. Furthermore, engaging patients’ carers further widened accessibility to the virtual ward model, including for patients who were non-English speakers. Our focus on education to build digital familiarity, skills and confidence was fundamental to the successful engagement of patients in remote monitoring. Additionally, a low threshold to offer additional support via more frequent telephone checks or provision of pulse oximeters where necessary ensured equitable access to all virtual ward patients. These methods have also been reported elsewhere as fundamental approaches to addressing the barriers to digital inclusivity [20].

There are several limitations to our work, particularly given this work was undertaken as a service improvement project rather than as a formal research study. Our AF virtual ward was implemented across 2 London hospitals and therefore the results may not be generalisable to the wider population. The study is observational in nature and no matched control group was recruited, such that confounding factors may have influenced the results obtained. Furthermore, the sample size of the patient cohort was small and the study is underpowered to draw statistically meaningful conclusions. Future larger prospective studies of AF virtual wards should aim to be randomised and include a matched comparator cohort to definitively evaluate the clinical benefit of the intervention. We also acknowledge that the follow up duration is not long enough to determine if patients sustain their good health over a longer time span. We are careful to make any comparisons against the unmatched retrospective patient cohort as these are also prone to the effects of confounding and therefore cannot be statistically valid. A formal cost effectiveness analysis has not been undertaken as part of this study, and the cost of an inpatient admission provided is an estimate and based on several assumptions from the extrapolation of historical admissions data.

## Conclusion

In this study, we present a novel AF virtual ward model, which can be implemented at relatively low cost and with wider accessibility using smartphone-enabled remote monitoring via a centralised monitoring hub. Our findings indicate that our virtual ward model enabled patients to be effectively triaged remotely, allowed health deteriorations to be recognised early, ensuring patients received timely medical attention or emergency care, and reduced the likelihood of subsequent ED reattendances on discharge. We also demonstrate the potential cost effectiveness of our model through readmission avoidance and early supported discharge of stable hospitalised AF patients, who might otherwise have had a longer inpatient stay. Larger scale prospective studies and robust clinical trials are needed to evaluate the long-term safety and clinical utility of AF virtual wards beyond conventional care. This pilot study provides valuable insights into the promise of integrating digital health and remote monitoring into AF management via a virtual ward model.

## Supporting information

S1 Appendix AF care plan. (PDF)

S2 Appendix Hospitalised AF Patient Cohort. (CSV)

S3 Appendix AF Virtual Ward Cohort. (CSV)

S4 Appendix Measurements From Remote Monitoring Application. (CSV)

S5 Appendix Patient Satisfaction Survey Data From Remote Monitoring Hub. (CSV)
