# Supplementary material for: Insights from a single centre implementation of a digitally-enabled atrial fibrillation virtual ward
Source: PLOS Digit Health. 2024 Mar 20;3(3):e0000475. doi: 10.1371/journal.pdig.0000475 (PMC10954132; doi:10.1371/journal.pdig.0000475)
Supplement: S1 Appendix — (PDF) [file pdig.0000475.s001.pdf]

Atrial Fibrillation (AF) Care Plan

Monitoring

Symptom Tracker

Populated from FibriCheck, includes severity (mild-severe)

- Palpitations (sensation of your heart racing or skipping beats)
- Dizziness
- Breathlessness
- Chest pain
- Tiredness
- Blackouts

Measurements

Populated from FibriCheck

- Heart rate (bpm)
- Heart rhythm

Action plan

You have been diagnosed with atrial fibrillation (AF), which is a disturbance of your heart rhythm.

We would like you to monitor your heart rate, heart rhythm and symptoms at home over the next two weeks using the **FibriCheck app** on your smartphone, with support from our **Remote Monitoring Hub**.

The aims of this plan are:

- To ensure your medications are best optimised for your atrial fibrillation
- To better manage any symptoms
- To detect any changes in your condition that may warrant earlier medical attention
- To support and educate you regarding your condition

How do I activate the FibriCheck app on my smartphone?

You will need a smartphone and an email address.

1. Download the FibriCheck app on your smartphone (via the Apple App Store or Google Play Store)
2. Register onto the FibriCheck platform by creating an account. You will need to provide an email address, which should be the same as the email address used for registering onto the Care Information Exchange.

3. Activate your invitation code. Firstly tap “Start Measurement”. You can then scan the QR code within the app using the phone camera, or alternatively enter in the code invitation code provided on the flyer.
4. Go to “Settings” and tap on “Connected apps”. Then select “Patient Knows Best”. Tick the box to share your FibriCheck account with the Care Information Exchange. Login to Care Information Exchange and then tap “Back to app” and then “Open in FibriCheck” to start measuring.

How do I make measurements and record my symptoms?

STEP 1

Access the FibriCheck app

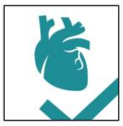

STEP 2

Take a measurement by placing your finger over the camera as shown

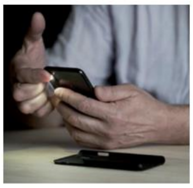

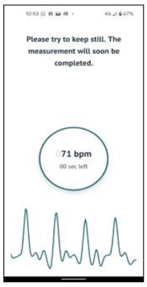

STEP 3

Record your symptoms on the next page

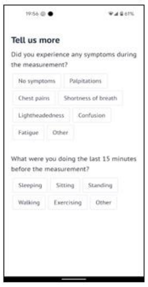

STEP 4

Your measurements and symptoms will be uploaded onto Care Information Exchange for review

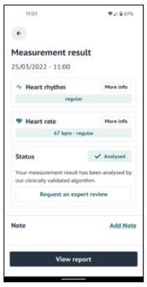

You can perform heart rate and rhythm measurements by pressing the “Start measurement” button. Place your finger on your smartphone camera as shown above. After a 60-second measurement, you will be asked to input your symptoms. These data will be shared onto the Care Information Exchange and reviewed by the remote monitoring team.

Please take a measurement at least twice a day for seven consecutive days. You may be asked by a member of the remote monitoring team to continue taking measurements for up to 14 days.

All measurements should be taken while you are resting or sat down. Your first daily measurement should ideally be performed **between 9-10am** and your second daily measurement **between 4-6pm.** This will enable the remote monitoring team to review your measurements and symptom entries in a timely manner.

You may wish to record additional measurements outside of these times, such as if you are experiencing symptoms, but they may not be reviewed until the following day. Please refer to advice in the **RED-AMBER-GREEN** section for further support about what to do if you notice any changes in your symptoms.

Who can I contact for further support?

The remote monitoring hub can be contacted between **9am-7pm daily** (including weekends and bank holidays) for assistance, support and to discuss any other concerns. They can be contacted directly on the following number: **07385950852**.

A member of the team will contact you following your visit to the Ambulatory Emergency Care (AEC) Unit to provide guidance, support and any troubleshooting assistance.

If your heart rate is too high or too low, or you have **"RED FLAG"** symptoms, you will be contacted by our remote monitoring team. In this case, you may be asked to attend either the AEC Unit or the emergency department depending on how unwell you are deemed to be.

You may also be contacted by the remote monitoring hub if you miss several measurements for further support.

What will happen after your remote monitoring period?

A member of the team will contact you at the end of the remote monitoring period to advise you on your ongoing care.

The team will review the information you provide and if needed, you may have a future appointment with a cardiologist and additional tests.

If your heart rate and symptoms are well-controlled, you will be discharged back to your GP provided you have no symptoms of concern.

If your heart rate is not well-controlled, you may have an appointment to be reviewed in the AEC Unit and/or be referred to the cardiology clinic to have additional tests organised.

Ambulatory care emergency (AEC) team responsibilities:

- Explain your diagnosis clearly and provide further information and advice
- Enrol you onto the Care Information Exchange and help you register on to the AF care plan
- Provide a QR code to enable you to activate the FibriCheck app
- Provide a QR code for sharing your clinical records on Care Information Exchange with the remote monitoring team
- Refer you to the digital monitoring hub
- Commence appropriate medications for your atrial fibrillation
- Review your atrial fibrillation treatment if your symptoms or heart rate are poorly controlled
- Consider referral to cardiology to consider other possible treatments if indicated
- Advise you on when to seek urgent medical attention

Remote monitoring team responsibilities:

- Provide troubleshooting advice for using the FibriCheck app
- Monitor your heart rate, rhythm and symptoms uploaded via the FibriCheck app onto the Care Information Exchange
- Contact you in the event of abnormal measurements or symptoms of concern to assess you
- Advise you whether to seek urgent medical attention

Recommendations to patients:

- Please ensure you continue to take measurements and input your symptoms twice daily over the monitoring period. In order to do this, you will need to have the FibriCheck app downloaded and be able to access the Care Information Exchange online.
- Take your medications as prescribed. If you think any of your tablets are disagreeing with you, please inform the AEC team or your GP who can investigate.
- If you experience difficulty in breathing, severe chest pain, severe dizziness or blackouts, seek urgent medical attention.
- Don't smoke.
- Please limit your alcohol intake to the recommended weekly limit.
- Try to be as active as is safely possible.
- Eat a varied healthy diet.

This care plan is to help you, or a carer self-manage your atrial fibrillation. You can use this plan as your online resource folder. You can access online links, watch a video from British Heart Foundation and also track symptoms.

Please click the button to add your: Diagnosis

Please click the button to add any Allergies you may have: Allergies

Please keep your medications updated, you can do that in your record: Medications

What is atrial fibrillation?
https://www.youtube.com/watch?v=ezTEc6GwLNs

Online resources:

https://www.bhf.org.uk/informationsupport/conditions/atrial-fibrillation
https://www.heartrhythmalliance.org/aa/uk/atrial-fibrillation

Other useful links:

Diagnoses

Go to Diagnoses

Any diagnoses in the patients record will show here

Allergies and adverse reactions

Go to Allergies

Any allergies in the patients record will show here

Medications

Go to Medications

Any medications in the patients record will show here

Green

GREEN - KEEP WATCH

Please continue to monitor your heart rate and heart rhythm twice daily on the FibriCheck App for at least 1 week.

Please also monitor for any change in your symptoms, which can be input into the FibriCheck App after you have made a measurement.

Your heart rate should be between 60 and 110. You agree with the statements below:

How am I?

You are asymptomatic. You do not experience any breathlessness, chest pain, dizziness or black outs.

You may experience occasional palpitations but you feel well and these are not uncomfortable for you.

What should I do?

There is no need for you to seek medical attention.

The Remote Monitoring Team can be contacted directly for advice **(9am-7pm)** via the telephone number in the contact information section.

Amber

AMBER - KEEP WATCH

Please continue to monitor your heart rate and heart rhythm twice daily on the FibriCheck App for 2 weeks.

Please also monitor for any change in your symptoms, which can be input into the FibriCheck App after you have made a measurement.

Your heart rate was between 50-60 or between 110-120 for 2 or more measurements. For all other measurements, your heart rate was between 60 and 110. You agree with the statements below:

How am I?

You are no more breathless than at the time of your initial Ambulatory Emergency Care (AEC) appointment or at the time of your discharge from hospital.

You may have experienced palpitations, dizzy spells or mild chest pain.

You have not had any blackouts

You have not developed severe or incapacitating dizziness.

What should I do?

You will be contacted by the Remote Monitoring Team in-hours (9am-7pm) who will assess you and discuss any concerns with your medical team.

Following an assessment from the Remote Monitoring Team, you may be contacted by a member of the Ambulatory Emergency Care (AEC) team to organise a review of your symptoms, heart rate control and medications. Depending on your symptoms, you may be referred for a cardiology opinion to consider alternative treatments.

The Remote Monitoring Team can be contacted directly for advice **(9am-7pm)** via the telephone number in the contact information section.

Red

RED - TAKE ACTION

Please continue to monitor your heart rate and heart rhythm twice daily on the FibriCheck App for 2 weeks.

Please also monitor for any change in your symptoms, which can be input into the FibriCheck App after you have made a measurement.

Your heart rate was below 50 or above 120 OR you have had any of the below symptoms:

How am I?

Your breathlessness is worse than your initial Ambulatory Emergency Care (AEC) appointment or at the time of your discharge from hospital.

Your usual exercise capacity is limited because you are getting breathless.

You have had an episode of moderate or severe chest pain

You have had a black out

You have experienced severe or incapacitating dizziness.

What should I do?

Please contact the Remote Monitoring Team in-hours (9am-7pm) who will assess you and discuss any concerns with your medical team. The Remote Monitoring Team may also contact you directly in-hours to assess you. You may be asked to attend either the AEC Unit or the emergency department depending on how unwell you are deemed to be. You may be referred for a cardiology opinion to consider alternative treatments.

Out of hours, consider urgent advice from your GP or 111 if you have any of the highlighted red flag symptoms.

If you feel unwell at any time or are having ongoing severe chest pain, severe breathlessness, severe dizziness, or have had a blackout. Go directly to your nearest emergency department or call 999.

Contact information

Remote monitoring hub **(9am – 7pm)** – **07385950852** (for help and advice whilst actively monitoring)

Following your monitoring interval, if you require further help/support, please contact your GP/111 or 999 in the event of an emergency.
